# What Do Chinese Families With Depressed Adolescents Find Helpful in Family Therapy? A Qualitative Study

**DOI:** 10.3389/fpsyg.2020.01318

**Published:** 2020-06-30

**Authors:** Liang Liu, Jiajia Wu, Jing Wang, Yan Wang, Yuezhou Tong, Congcong Ge, Yanbo Wang

**Affiliations:** ^1^Shanghai Pudong New Area Mental Health Center, Tongji University School of Medicine, Shanghai, China; ^2^Department of Clinical Psychology, Shanghai East Hospital, Tongji University School of Medicine, Shanghai, China; ^3^Department of Psychology, School of Humanities, Tongji University, Shanghai, China; ^4^Division of Medical Humanities and Behavioral Sciences, Tongji University School of Medicine, Shanghai, China

**Keywords:** adolescent depression, family therapy, qualitative, subjective experience, therapeutic factors

## Abstract

Despite research supporting the efficacy of family therapy for adolescent depression, little research has been done to clarify the therapeutic variables that Chinese families with depressed adolescents consider helpful in family therapy. This study explored depressed Chinese adolescents’ and their parents’ perceptions of the factors promoting improvement in family therapy. Twelve Chinese families with one adolescent child fulfilling the criteria for major depressive disorder were recruited. A total of 134 family therapy sessions were conducted by four therapists. After therapy, semi-structured interviews about the clients’ perceptions of the helpful and effective aspects of therapy were conducted. Thematic analysis was used to analyze the transcribed dialogues. Five overarching themes emerged: *factors supporting therapeutic alliance formation, the therapist’s systemic attitude, systemic therapy direction, effective systemic therapy techniques*, and *strategies for dealing with current issues.* A trustworthy therapy alliance with a competent and supportive therapist helped depressed adolescents improve by facilitating the expression of their emotions; exploring family interactions, beliefs, and emotion flows related to their symptoms; promoting their self-development; and activating their resources. The families valued the respectfulness of the therapists and their collaborative and non-authoritative approach. Adolescents’ academic difficulties and crises received special attention. The possible clinical implementations of our findings in the design of family therapy strategies for depressed Chinese adolescents are discussed.

## Introduction

Adolescents (13–18 years old) are one of the highest risk groups for depressive disorder, with the total prevalence of depression risk in children and adolescents reaching 23.9% (Wang et al., 2016). The complications of depression in adolescents are serious and closely linked with negative outcomes in adulthood, including higher rates of major depression, anxiety disorder, illicit substance abuse/dependence, and intimate partner violence victimization (McLeod et al., 2016). Thus, there is an urgent need to identify effective and targeted clinical treatments for adolescent depression.

There is emerging evidence of the efficacy of family therapy for adolescent depression (Zhang and Dong, 2013; Li et al., 2016). Family therapy is a systemic psychotherapy approach that focuses on assessing and improving the interpersonal processes in clients’ families, and in turn promoting the remission of symptoms (Hu, 2010; Sexton, 2019). Prior studies have demonstrated that among depressed adolescents from different cultures, family therapy can promote a decrease in depression symptoms, shorten the recovery time, and improve patients’ family function and social performance (Keitner et al., 2009; Zhang and Dong, 2013; Li et al., 2016).

Nevertheless, little research has been done to clarify which therapeutic methods, techniques, or factors Chinese families with depressed adolescents identify as helpful in family therapy themselves. This research gap has the potential to generate problems, e.g., therapists may devote much energy to developing therapeutic strategies that might ultimately be indicated to be ineffective. Such practices could lead to unnecessary consumption of clinicians’ and clients’ time and might obstruct the application and popularization of family therapy for adolescent depression (Liu et al., 2013).

There have been reports on the potential factors affecting adult and adolescent clients’ experience of psychotherapy for other individual psychotherapeutic approaches, such as psychoanalysis, cognitive behavioral therapy (CBT), interpersonal psychotherapy, etc. Generally, the variables affecting clients’ responses to psychotherapy can be grouped into the following categories: (1) therapist competence, (2) therapeutic alliance and setting, (3) pretreatment characteristics of clients, and (4) psychotherapy techniques and strategies.

First, in individual therapy for adults and adolescents with different clinical complaints, the prior literature has suggested a positive correlation between client satisfaction with therapy and therapist competence in terms of empathy, theoretical interpretation, psychoanalytic investigation, clear communication, respect, warmth, positive interpersonal skills, and therapist authenticity (Despland et al., 2009; Anderson and Strupp, 2015; Anderson et al., 2016; Levitt et al., 2016; Schottke et al., 2017). The depressed adolescents who accepted psychodynamic individual therapy in Lovgren et al.’s (2019) study highly valued the therapist’s confidence and professional experience.

Second, previous research has found that the setting of psychotherapy, including the involvement of parents, a shorter intake time and a moderate frequency and duration of sessions, was associated with better outcomes and less dropout in psychotherapy for adolescents with depression (Dardas et al., 2017; Lovgren et al., 2019). Additionally, previous studies have suggested that stable, collaborative, supportive, and trustworthy relationships between therapists and clients in family and individual therapy not only help to reduce the dropout rate from therapy, but also promote clients’ improvement (Robbins et al., 2006; Levitt et al., 2016; Yoo et al., 2016; Lovgren et al., 2019). For example, Feder and Diamond (2016) found that in attachment-based family therapy for suicidal and depressed adolescents, the strength of the parent-therapist alliance predicted parents’ subsequent attachment-promoting behaviors.

Third, the pretreatment patient characteristics contributing to poorer outcomes usually include more severe depressive symptoms, less comorbid anxiety, and refusal of medication. Empirical correlations between these pretreatment characteristics and the client’s response to psychotherapy have been partially established for adult and adolescent depression (Lopes et al., 2015; Spinelli et al., 2016).

Fourth, reports of the association of therapy skills and strategies with therapy outcomes have varied across different therapy approaches for depressive disorder. In family therapy, techniques that have been proposed to be effective include posing circular questions, creating genograms, reframing, making metaphors, expressing acknowledgment, using reflecting teams, playing video games, reformulating, and giving feedback (Sundet, 2011; de Leon, 2018; Earl, 2018; Larner, 2018; North et al., 2018). In attachment-based family therapy for depressed adolescents, previous research reported that encouraging clients’ affect, focusing on adolescents’ unmet attachment/identity needs, relational reframing, and focusing on clients’ primary adaptive emotions allowed therapists to promote adolescents’ productive emotional processing and improve the quality of their attachment with their parents, in turn promoting relief of their depressive symptoms (Diamond et al., 2002; Ewing et al., 2015; Tsvieli et al., 2019). In psychodynamic therapy of adolescent depression, clients have considered self-exploration and discussion of issues in their daily lives to be helpful (Lovgren et al., 2019).

This review of previous studies suggests an absence of studies exploring the therapeutic variables that Chinese families with depressed adolescents identify as helpful in family therapy. Compared with Western culture, Chinese culture places a greater emphasis on the Confucian values of filial piety and children’s obedience to their parents’ authority (Liu et al., 2014). Furthermore, China’s one-child policy may make parents pay more attention to their children, and the parent-child relationship might be more entangled. This implies that Chinese adolescents may be at greater risk of being triangulated into their parents’ marriage conflicts and affective processes, and may experience more expectations and control from their families (Mota and Matos, 2014; Wang et al., 2014; Hollenstein et al., 2015; Fan, 2016; Kloep et al., 2016). Additionally, Chinese adolescents may experience greater academic pressure (Hou and Chen, 2016). Previous research implies an association between these culture-related factors and the occurrence and maintenance of depression in Chinese adolescents (Wang et al., 2013, 2014; Fan, 2016; Hou and Chen, 2016). These differences could motivate Chinese family therapists to develop more culture-targeted interventions for depressed adolescents, and might in turn make clients’ subjective experience of the therapy process different from that of their Western counterparts. However, which tailored interventions employed by Chinese therapists are considered useful by depressive adolescents’ families remains unclear.

To date, nearly all the studies that have been related to family therapy in China have been clinical trials focusing solely on changes in symptoms and the family environment after therapy or on therapists’ personal experiences and comments (Zhao and Xuan, 1999; Zhang and Dong, 2013; Li et al., 2016). Although Liu et al. (2013, 2014) conducted a qualitative analysis on the process of Chinese family therapy for adolescent and adult clients with various clinical complains and summarized the therapeutic strategies and techniques that Chinese therapists employed, the researchers focused only on what therapists did during regular daily therapy processes without exploring clients’ opinions about the therapy. Their studies also did not attempt to distinguish the sessions that were identified by Chinese patients as successful from those that were considered less useful. Similarly, van der Pol et al. (2019) summarized common elements of systemic therapy for adolescents with disruptive behavior problems based on the results of prior research. Levitt et al. (2016) conducted a qualitative meta-analysis on 109 qualitative studies examining clients’ experiences within adult individual psychotherapy and identified five clusters of factors: pattern identification, narrative reconstruction, developing self-awareness, collaborative and professional therapy structure, and recognition of clients’ needs and agency. However, their study did not focus specifically on what constitutes good therapy for Chinese families with depressed adolescents.

the aim of this qualitative study is to explore (1) depressed Chinese adolescents’ and their families’ subjective experiences of family therapy and (2) the therapy-related factors that adolescents and their families identify as helpful for the remission of symptoms. Because this was an exploratory study on Chinese families’ subjective experience of family therapy, as suggested by Levitt et al. (2016), a qualitative research approach was used to explore and clarify the clients’ opinions.

## Materials and Methods

### Participants

Twelve families were recruited between January 2018 and February 2019 from the mental health hospitals of Tongji University, Shanghai, China. All the families had an adolescent child (16–18 years old) diagnosed with *major depressive disorder* according to the criteria of the *Diagnostic and Statistical Manual of Mental Disorders*-5 (DSM-5). The inclusion criteria were as follows: the adolescent’s depression was *first-episode depression* with no *remission* (Richards, 2011); both parents participated in family therapy with the child; all family members participating in the therapy agreed to attend the interview after the therapy; and the parents and children had adequate cognitive abilities to provide accounts of their experiences.

All the families were one-child families. The duration of their depressive episode ranged from 6 months to 1 year. All the children, 10 females and two males, in the participants’ families were senior high school students. The participants’ families agreed to participate in family therapy conducted by four therapists, three of whom were male and one of whom was female. All the therapists were psychiatrists who had undergone long-term structural (Minuchin et al., 2013) and Milan systemic family therapy training (Selvini et al., 1980), with an average length of training of 10.25 years. The average age of the therapists was 38 years, and the average time they had been practicing was 10 years. The family therapy sessions were conducted every 2–3 weeks for each family according to their needs and schedules. The total length of the psychotherapy was 5–6 months for each family, and the total number of therapy sessions ranged from 10 to 11. The duration of each session was approximately 1.5 h. In addition, among the 12 adolescents, nine received antidepressant medication and family therapy, and the remaining three received family therapy only.

### Procedure

The participants were recruited from the outpatient department of the hospital. Potential participants who met the inclusion criteria were identified when they first visited their psychiatrists. They were then contacted by a member of the research team through email or by phone. The purpose, content, process, and principle of confidentiality of the study were introduced to the potential participants in accordance with standardized instructions. Then, informed consent was obtained if they agreed to participate in the study. The families’ demographic data were also gathered at that time. After the families finished the entire family therapy process, they were invited to participate in an interview as a family (father, mother, and child). In total, 31 potential participant families were identified. Fourteen of them refused to participate. Among the remaining 17 families who were enrolled in the study, give quit due to time limits, and the remaining 12 families completed the interviews. The interviews were conducted in a private room, and the duration of each interview was approximately 90 min. All four researchers who carried out the interviews had undergone training in qualitative research for at least 6 months. The interviews were audio-recorded with the participants’ permission and then transcribed verbatim. Field notes including the families’ demographic data, the main content of the interviews, and the self-reflections of the researchers were also made before, during, and after the interviews. Each interview was conducted until the experiences of the family members in family therapy were well-explained and no new information was available. When no new information was forthcoming, saturation was considered to have been reached.

The following semi-structured interview guide was used during the interviews:

1. Could you please tell me how you feel after this treatment?
2. What did your doctor say (or do or discuss with you) that helped you during the treatment?
3. How would you rate the therapy sessions regarding their usefulness? Why is that?
4. What touched you the most during the conversation?
5. What other aspects did you find helpful (regarding your family relationships or your own state of mind)?
6. What did you find less satisfying about the treatment? If so, what could be done in the next treatment that would make you feel better?

The research proposal received ethical approval from the ethics committee of Tongji University as well as the Shanghai Pudong New Area Mental Health Center. Participants were informed of the purpose of the research, after which written informed consent was obtained from the adolescents and their parents for their participation in this study. All the members of the adolescents’ families supported each other’s participation in the therapy. Written informed consent was also obtained from the parents of the adolescents for the publication of their research data. To protect the privacy of the participants, we use “F1, F2… F12” to represent the different families when quoting their conversations.

### Data Analysis

**FIGURE 1:**
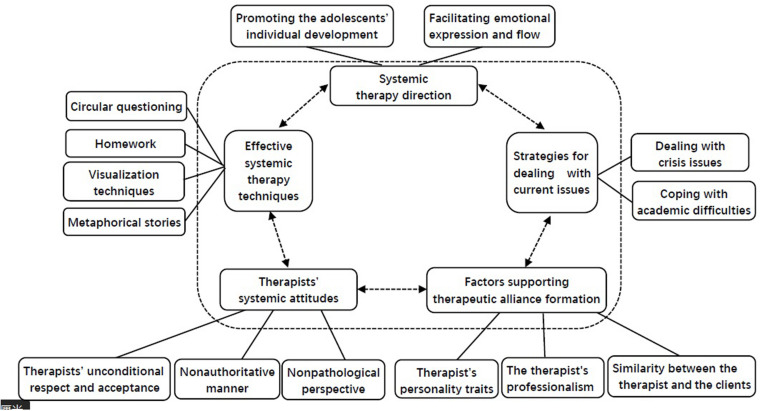
Map of thematic analysis.

Thematic analysis was used to analyze the transcribed data (Braun and Clarke, 2006). Thematic analysis is a method that allows the identification, analysis, and reporting of patterns (themes) within data. It is compatible with both essentialist and constructionist paradigms within psychology. Because of its theoretical freedom, thematic analysis is a flexible and useful research tool that can potentially provide a rich and detailed yet complex account of the data. Moreover, thematic analysis has been widely used in studies on psychotherapy process research (Liu and Zhao, 2009; Liu et al., 2013, 2014). Analysis of the dataset was carried out following the guidance provided by Braun and Clarke (2006). The first step was reading and rereading the data and noting initial ideas. The second step involved coding interesting features of the data in a systematic fashion across the entire dataset and collating the data that were relevant to each code. The third step was collating the codes into potential themes and gathering all the data relevant to each potential theme. The fourth step was examining how the themes worked in relation to the coded extracts and the entire dataset and then generating a thematic map of the analysis. Finally, the accepted themes were refined, and a thematic map was finalized for the report (see Figure 1). The initial codes and potential themes were developed by the first and corresponding authors, both of whom have been conducting psychotherapy for at least 10 years and have more than 9 years of experience in qualitative research. Then, the first and corresponding authors reviewed and refined the themes through an iterative process until a consensus was reached on the final thematic map.

## Findings

Five main themes were developed, each with subthemes. The finalized thematic map is shown in Figure 1. The first theme reflected the *factors supporting therapeutic alliance formation*; the second theme focused on the *therapist’s systemic attitude*; the third theme covered the *systemic therapy direction*; the fourth theme displayed *effective systemic family therapy techniques*; and the last theme concerned *strategies for dealing with current issues*.

### Theme 1: Factors Supporting Therapeutic Alliance Formation

The relationship formed between the depressed adolescents’ families and the therapists was found to be an essential element of effective psychotherapy. A key feature of the relationship-building capability was therapeutic alliance formation. Some of the participating families emphasized that several factors helped them devote themselves fully to their psychotherapy. According to their views, therapists’ characteristics (such as personality traits and professionalism) seemed to be crucial to the formation of a good therapeutic alliance. In addition, similarities between the therapist and the clients also influenced early relationship building.

#### Subtheme 1: The Therapist’s Personality Traits

Four families (F1, F3, F5, F6) mentioned therapists’ personality traits, including their patience, affinity, warmth, and humor, as the overarching factors that helped the family members to continue to participate in psychotherapy. F3 provides an example:

The doctor was warm and humorous. He said that I was part of the 5% of the world’s depressed population who could tell my tragic experience in a funny way. He hoped I could be a funny comedian. These words made me feel warm and happy.-F3 (daughter)

The doctor looked very friendly and nice. He is totally different from those clinicians we met before, who looked unconcerned and passive. -F3 (mother)

#### Subtheme 2: The Therapist’s Professionalism

Professionalism referred to the therapists’ competency in handling and providing information, their professional training background as psychiatrists, and their professional meticulousness and sensitivity. In the sessions, the clients’ families were often confused and curious about the disease diagnosis, the etiology of the disease, and the curative effect of drugs. Professional and reasonable explanations from the therapist provided them with powerful informational support. Participants from four families (F2, F6, F7, F9) emphasized that the therapist’s professional background as an authoritative psychiatrist provided a sense of trust and stability in the therapeutic alliance. Members of F2 and F6 stated the following:

The doctor told me that I was depressed and needed to take medicine and psychotherapy. Finally, I knew what was happening to me, and my symptoms were finally explained. -F2 (daughter)

I feel that my psychiatrist is great. He made me understand what caused my daughter’s problem. It is more effective than traditional Chinese medicine. I used to be desperate, but now I have confidence. -F6 (mother)

Two families (F3 and F5) highly valued their therapist’s ability to summarize and sort out complex information. It enabled them to trust the therapist and experience satisfactory treatment outcomes. This is illustrated in the following participant statements:

The doctor was very good at sorting through my jumbled information and quickly finding the source of the problem. He was also really professional. -F3 (daughter)

He could quickly grasp a point from the professional side and helped us communicate with our daughter. He could also influence my thinking and helped me consider from another perspective, which was very good. -F5 (mother)

In addition, the adolescents from 10 families mentioned that the integrity and meticulousness of the therapists and their high sensitivity to the clients’ needs met their standard of professionalism. These were important factors motivating the clients to adhere to the therapy and made them feel warm.

At first, I thought he would only ask questions casually and generally, but later, I found that he even asked about a lot of details about the relationships in our family. I was touched by his integrity and meticulousness. I felt that the clinical work of doctors was to understand me and my inner thoughts and emotions better. -F1 (daughter)

#### Subtheme 3: Similarity Between the Therapist and the Clients

Some clients (F3, F10, F11) preferred to choose therapists who were similar to them. This similarity motivated them, to a certain extent, to actively join the therapeutic alliance. Taking the adolescents in F10 and F11 as examples, the similarities in age, cultural background, and worldview between them and their therapists were the main reasons they participated in the therapy. As the son of F10 stated:

When I came here for the first time, I felt that the doctor was not very old, and his personality was similar to mine. I felt that he was not an authority; there was no great distance between him and me. I felt that he was a person who was very easy to communicate with. -F10 (son)

Similar geographic origins and cultural backgrounds also affected the trust of the clients in the therapist. The mother of F3 was from the same hometown as her therapist, which brought her closer to and increased her trust in the therapist. She stated the following:

I am very pleased to talk with him (Therapist A), not only because of his ability. It is also because he comes from the same province as us. We have similar ideas and habits. This similarity in our background is important. -F3 (mother)

### Theme 2: Therapists’ Systemic Attitudes

The clients’ families mentioned helpful therapist attitudes that stimulated positive processes of change. These attitudes included the therapists’ *non-pathological perspective*, *non-authoritative manner*, and *unconditional respect and acceptance*.

#### Subtheme 1: Non-pathological Perspective

During the therapy, despite the diagnosis of depression, the adolescents were not treated as disabled or shameful by the therapists. The adolescents’ abnormal behavior or problems that were previously seen as unacceptable by their families were endowed with positive meaning and reinterpretations. To some extent, this perspective alleviated the anxiety of the whole family and was highly valued by 11 families. As the parents of F5 and F11 described,

I think my daughter was very tightly wound. When I came here, the doctor didn’t treat her as a patient. He said that normal people also had emotional fluctuations. These words made her feel more relaxed. -F5 (mother)

I used to think that certain behaviors were pathological, and I always wanted to reverse them. The doctor showed me that some of my child’s behaviors were not pathological, but just an unhealthy lifestyle. Now they are acceptable. -F11 (father)

Additionally, discussion between the therapist and the client’s family about the function of the symptoms broadened the families’ perspectives on their problems. Shifting the focus from the problems to the strengths of the clients made the family members see that they had more resources to cope with the current predicament. The mothers in F4 and F6 provide examples:

The doctor guided us to look at problems dialectically. Don’t take the idea that her ‘brain can’t work’ as an obstacle to the family or life, but see what positive aspects it can bring. It seems that her depression can bring a temporary calm to our family. -F6 (mother)

The non-pathological perspective also showed the therapists’ preferences for affirming family resources, shifting the focus from the problems to the strengths of the clients. For example, the mother of F4 described this approach as follows:

After all this treatment, I saw more of my daughter’s positive qualities and became less worried. -F4 (mother)

#### Subtheme 2: Non-authoritative Manner

Under traditional Chinese filial piety, parents and schoolteachers are generally regarded as unchallenged authorities. Children’s unconditional obedience to their parents and teachers is highly valued. However, most adolescents in our study expressed disagreement with this Chinese cultural tradition. Correspondingly, what the adolescents complained about most was that their parents always judged their worldview, criticized their lifestyle, pushed them to accept ideas that they disagreed with, and controlled their lives.

In contrast, the adolescents believed that the therapists in our study had a non-authoritative manner and talked with them on equal footing. They felt that their worldview, values, and lifestyle (except for their suicidal or violent tendencies) were accepted by the therapists. Their dialogue with the therapists was carried out in an equal, non-judgmental style full of mutual respect. Ten of the adolescents in this study highly appreciated the therapists’ position of a non-authority. This also brought the therapists and patients closer. As the son in F10 described,

*My former therapist, who was an authority, talked to me in a way that felt like my dad was talking to me, which made me feel distant and uncomfortable. But my current therapist, he always wears his typical smile, and I feel he is a person who is easy to communicate with and not authoritative. He never judges me. Our distance is suddenly narrowed.* -*F10 (Son)*

Moreover, the therapists also tried to improve family relationships by helping parents respect and accept certain lifestyles and values of their children. They encouraged the families to communicate with the adolescents in a non-authoritative manner. The father of F11 reported the following:

*The doctor says she doesn’t want to go out. It’s just her lifestyle. It doesn’t mean she’s sick. This explanation eased my anxiety. If she doesn’t feel like going out, don’t go out. I used to keep pushing her out. Now I don’t push her anymore, and the relationship between us has improved.* -*F11 (Father)*

#### Subtheme 3: Therapists’ Unconditional Respect and Acceptance

Ten families in our study also highly valued the therapist’s acceptance of and respect for their worldviews, emotions, feelings, expectations regarding the treatment plan, and desired pace for rehabilitation. First, the patients and families perceived that their emotions and worldviews were respected by their therapists. For example, the daughter of F7 said the following:

My parents have been trying to comfort me, telling me not to worry, as if my fears were wrong. That makes me feel embarrassed and guilty. However, my doctor told me, ‘You can worry; we can do something else to let go of that worry,’ and it made me feel comfortable; it’s like he was closer to my feelings.

The respect of the therapists for their clients was also reflected in the fact that they respected the client’s desired pace for therapy and rehabilitation without urging or controlling them. The doctors also helped the family members accept and respect the patients’ pace of recovery, which allowed parents to feel less anxious, as the son of F10 and the mother of F6 stated:

My doctor is the kind of guy who can make you feel understood and comfortable, no matter what your mood is. It’s like climbing stairs. If you speed up, he can follow you. If you slow down, he can also relax. He’ll always be at your pace. -F10 (son)

The doctor invited us to stand in the daughter’s position to consider. He said, ‘Let anxiety take place for a few days, it doesn’t matter.’ Before, we always felt anxious when we thought she was going to have an exam. Now, we feel it is fine to let her feel anxious for a few days because this is a normal emotion. -F6 (mother)

In the treatment, after conducting the risk assessment, the doctors usually made treatment decisions that fully respected the wishes of the patient and the family members, including regarding the choice of treatment method, whether or not to take medication, and the adjustment of drug dosages. As the mother of F8 shared:

The therapist respected our will. We could choose to take medication or not, but we just want to do therapy only. The doctor has helped us to arrange that. -F8 (mother)

### Theme 3: Systemic Therapy Direction

The identified patients in this study were all adolescents. Our analysis implied that the therapists in our study focused on promoting individual differentiation and helping family members build boundary consciousness. In addition, we found that many of the clients’ families were characterized by constant conflicts between family members and poor communication. Thus, promoting positive communication and emotional flow among family members was another major direction that clients mentioned promoted therapeutic progress.

#### Subtheme 1: Promoting Adolescents’ Individual Development

Self-differentiation and development are the main tasks of adolescents. All the families in our studies were one-child families. Our analysis demonstrated that too much of the parents’ attention was focused on the adolescents, most of whom were too enmeshed with their parents. Excessive attention from parents was a major feature of most of the families with ill children. In the sessions, adolescents’ individual development was first promoted by helping family members establish boundary awareness, as stated by the son of F10:

Mom, dad, and I should be a triangle. I used to be too close to mom. Now, they and I are still very close, but not as close as before. The two of them are closer to each other now. This feeling is ideal for me. After all, for boys at this stage, who wants to be so tied to their parents? -F10 (son).

Through the treatment, parents also felt it necessary to give children enough room for growth, as shown in the reflections of the mother of F3 and the father of F6:

The doctor told me to leave her alone and let her go completely, to give her some space, to let her play freely, it will be better; this point impressed me the most. -F3 (mother)

I may have arranged too many things for my daughter. Before, my daughter would come to me for everything, and I always satisfied her needs. In fact, this deprived her of the ability to do things herself. After the therapy, I just realized that I should change -F6 (father)

#### Subtheme 2: Facilitating Emotional Expression and Flow

In 10 families in our study, there was a long-term backlog of negative emotions that could not be effectively expressed. Therapists’ effective questions, active listening, and timely feedback allowed the families to find an outlet for emotional venting. The members of F6 and F8 provide examples:

He listened carefully to everything I said; I feel very satisfied. I think my parents also said what they wanted to say, thanks to him. -F6 (daughter)

It’s something like this. People are sitting here talking. It felt like he asked some very simple questions to guide me and then asked me to talk, and then I cried. I don’t know why he was so successful in blowing me up. -F8 (daughter)

Additionally, the use of painting to express emotions was novel and effective for the daughter in F3. She is quoted below:

The doctor allowed me to draw, which feels great. I painted pictures that made people feel extremely depressed, like the world was falling down, but he didn’t get scared away. -F3 (daughter)

Moreover, in the interviews, long-term poor communication between family members was found to be common. The therapy provided the families a space for communication. The therapists’ circular questioning made it easier to communicate, as the son from F12 stated:

I have a lot of conflict with my father, and he doesn’t understand me. Sometimes the doctor asks me or sometimes my dad, ‘Do you agree?’ He was listening as I was talking to him. It was a type of indirect communication and I think it is very useful. -F12 (son)

Throughout the therapy process, clients experienced that the therapists continued to help them express their emotions to each other and teach them effective expression methods. Take two families as an example:

At first, we kept a lot of things bottled up. At my age, I don’t like to communicate with my parents very much. When I first got to the clinician (Therapist D), I didn’t want to talk about it. However, the doctor encouraged me to talk and helped me to clarify my true wishes. At the end, you will reach a breaking point and talk it all out. My dad was so confused after he listened. -F10 (son)

The doctor suggests that I listen to her when she’s upset, to not rush to give advice. If I give advice, she won’t listen to it. It will only hurt her more. -F3 (mother)

### Theme 4: Effective Systemic Therapy Techniques

Some of the families mentioned *effective systemic therapy techniques* that they thought contributed to their understanding and change process. The techniques mentioned by the families included *circular questioning, homework, visualization techniques*, and *metaphorical stories.* Table 1 presents the application of various techniques and descriptions of patients’ experiences of them. Among them, *homework* was the most widely used technique and mainly took the form of *listing the positive characteristics of and changes in family members*, *conducting paradoxical interventions, holding family meetings, recording personal emotions, making future plans*, and *completing once- or twice-per-day homework*. *Visualization techniques* included describing the patient’s distress and emotions with objects, drawing diagrams of relationships, and representing family relationships with small objects.

**TABLE 1:** Effective systemic therapy techniques.

**Technique examples**	**Family**	**Extracts**
Circular questioning	F3, F9, F10, F11, F12	*The doctor asked a lot of interesting questions. He asked my father ‘Could you guess what your son thinks of your relationship with your wife,’ or ‘What kind of suggestions do you think your son would like you to give him?’ These questions were very enlightening and helped us think from the perspectives of the others. -F12 (son)*
Homework	All families	
– listing the positive characteristics and changes in family members		*The doctor asked me and her mother to write down our 10 favorite things my daughter has done. Her mother wrote 30 and suddenly realized how good her daughter was. -F1 (father)*
– conducting paradoxical interventions		*The doctor told me to choose to be anxious on odd days but not on even days. The result was that I did not feel anxious when it was the day to be anxious. -F6 (father)*
Visualization techniques	F6, F9, F10, F12	*The therapist referred to the water glass as my mother’s depression. Originally, the glass was in my hand, and I wanted to take care of my mother’s feelings, but now, I need to give this glass to my father, to let my father ease my mom’s mood while I take it easy so that the relationship between them will be better. Maybe at that time, my dad also felt something, and now their relationship is very good. -F10 (son)*
Metaphorical stories	F3, F9, F11	*The doctor told me a story about a little girl who wanted to bring something beautiful to the world, so she spread flower seeds everywhere and produced beautiful flowers. Then, the doctor said to me that I also want to bring some good to the world, which makes sense to me. -F9 (daughter)*

### Theme 5: Strategies for Dealing With Current Issues

The current issues of the adolescents with depression included crisis issues (e.g., self-harm and suicidal tendency) and academic pressure.

#### Subtheme 1: Dealing With Crisis Issues

Two families (F11, F1) mentioned therapy as a way to discourage self-harm or suicidal thoughts. The therapists dealt with suicidal issues by focusing on the adolescents’ pain; by exhibiting understanding, respect and non-judgment; by confronting suicidal thoughts and behaviors; and by building and maintaining solid alliance with the families. Two examples follow:

When my daughter said she had thoughts of dying, Dr. X said to her, ‘If so, as a doctor, I will try to stop you because I care about your safety. But if it was something else, I would encourage you, such as how to accept depression. If you have any more impulses to hurt yourself in the future, don’t hesitate to contact me or my assistant. We will try our best to help you.’ These days, my child has fewer thoughts about committing suicide. -F11 (father)

When the daughter of F1 was asked what impressed her the most during treatment, she said the following:

As for self-harm, the doctor made a small agreement with me that, as his patient, he would not let me do it. I acquiesced. He was trying to make sure I was safe as a patient. This makes me feel I have been cared for and respected. -F1 (daughter)

Interpersonal conflict at school was part of the reason for the pain felt by some of the adolescents in our study. The treatment gave them the inspiration to cope with interpersonal relationships, as described by the daughter of F9:

The doctor encouraged me to draw a picture to show my relationships with my classmates. Then she and I discussed about the possible reasons for my conflicts with them (classmates). She also helped me to think about strategies to improve my relationships with each classmate. I think it is useful. -F9 (daughter)

#### Subtheme 2: Coping With Academic Difficulties

All the teenagers in this study were senior high school students. Academic pressure, such as pressure to obtain higher scores and get into a good university, was the problem that created the most anxiety for almost all the families. In fact, 11 of the adolescents in this study refused to go to school or found the academic pressure exhausting. Our analysis showed that the therapists devoted a considerable amount of time to helping families deal with these academic difficulties, including the associated anxiety and frustrated emotions, and develop coping strategies. All the families expressed their approval of this intervention. Take F6 as an example:

The doctor suggested my parents stop forcing me to go to school all the time so that I can feel better. It’s my own business. It’s no use if they’re in a hurry. If they’re not, I’ll feel better. -F6 (daughter)

Her father and I were extremely concerned about whether she was able to go to school in September. After treatment, I feel it does not need to be mentioned every day, no matter whether she decides to suspend her studies or to learn, it will all be fine. If you let it go, she might go to school one day by herself. This idea makes me less anxious. -F6 (mother)

## Discussion

To our knowledge, this is the first study to explore depressed adolescents’ and their families’ subjective experiences of the process of family therapy in China. The first theme that emerged from the data concerned the factors affecting therapist-client alliance formation. Our results implied that within the context of family therapy for depressed adolescents, the quality of the therapeutic alliance played an important role in improving the clients’ experiences with therapy. This finding is consistent with reports from previous research that, within Chinese and Western culture, trustworthy therapeutic alliances not only constitute the basis of therapy, but also facilitate the process of change for adult and adolescent clients in family and individual therapy (Liu et al., 2013; Yoo et al., 2016; Lovgren et al., 2019).

Similar to previous research results, our results showed that among the factors affecting the therapeutic alliance, the therapists’ personality traits and professional competence, such as their mildness, stability, openness, patience, empathy, attention to clients, meticulousness, skill in collating and analyzing information, and ability to give theoretical explanations, made the clients trust them more (Despland et al., 2009; Sundet, 2011; Anderson and Strupp, 2015; Schottke et al., 2017; Lovgren et al., 2019). This finding may indicate that the form of treatment that depressed adolescents and families need most is humanized attention and acceptance from clinicians.

In addition, the families in our study attached great importance to the therapists’ professional backgrounds in psychiatry as well as their own similarities in age, worldview, and cultural background with the therapists. These themes may reflect the clients’ expectations about therapists, that is, that they should be credible, be professional, be trustworthy, have authority and medical knowledge, and be approachable. To a certain extent, this finding coincides with previous findings that working with experienced therapists and decreasing the distance between clients and therapists can optimize clients’ perceptions of psychotherapy (Sundet, 2011).

Regarding the second theme, *therapists’ systemic attitudes*, one main culture-related finding of our study was that the adolescents attached great importance to respect for and acceptance of their worldview and life attitudes by the therapists. They appreciated the non-authoritative and non-judgmental approach of the therapists. Some adolescents even directly expressed their disagreement with traditional Chinese filial piety. They hated when therapists took a position of authority and expected them to obey, just as their parents did. This finding is partially consistent with previous results indicating that too much emphasis on children’s one-way obedience to filial piety may contribute to more conflicts between Chinese parents and adolescents and might trigger more psychosomatic complaints in the children (Liu et al., 2014; Fan, 2016). It also implies that family therapists should show full respect for adolescents’ worldviews. However, the emergence of the theme *therapists’ professionalism* implied that sometimes the participants hoped that the therapists would provide necessary information and suggestions for their condition from a position of professional authority. This apparent contradiction suggests that therapists may have to maintain a balance in the treatment of adolescents with depression. They should maintain equal respect for young people in a non-authoritative position. At the same time, clinicians also need to be sensitive to their clients’ needs and give necessary informational support and suggestions in a cautious and timely manner.

Employing a *non-pathological perspective*, the therapists in our study treated the adolescents’ depression in a neutral manner with curiosity. They explored and expanded the families’ beliefs about their problems and helped them to develop new interpretations of the meaning of depressive symptoms. They also affirmed the families’ resources. Liu et al. analyzed the processes of 14 successful cases of family therapy and found that the interventions used most frequently by therapists were to *explore and develop new understandings*, *explore resources and empower*, and *explore and elicit solutions* (Liu et al., 2013). Sundet’s (2011) study demonstrated that clients appreciated family therapists’ efforts to identify more possibilities for improvement. Based on these previous findings and the results of our analysis, it is suggested that more attention be focused on resource-oriented interventions and the reframing of depression in therapy for Chinese families with depressed adolescents.

With respect to *therapists’ unconditional respect and acceptance*, a finding of our study is that Chinese families appreciated therapists’ willingness to negotiate with them in formulating treatment plans, including the choice for medication and psychotherapy. Especially for adolescent patients, when therapists showed respect for their own desired rhythm of rehabilitation, the topics they would like to discuss during treatment, and their expectations for therapy, their willingness to participate in therapy and make changes seemed stronger. This finding is partly consistent with findings showing that adolescents appreciated a non-authoritative therapeutic approach. This finding also suggests that for Chinese adolescents with depression, other people’s respect for their rhythm of rehabilitation and their willingness to engage in the treatment not only forms the foundation of psychotherapy but could also promote their rehabilitation (Sundet, 2011). This observation also corresponds to the principle of “neutrality” emphasized by family therapy (Liu and Zhao, 2009; Liu et al., 2013).

Among the subthemes of the third theme, *promoting the adolescents’ individual development* was especially valued by the participating families. As mentioned before, China’s one-child policy may lead to more enmeshment in the parent-child relationship. There might also be more interactions characterized by control and resistance to control in Chinese families, and children may be more often triangulated into parental conflicts and affective processes. This may hinder the differentiation and personal development of adolescents and might make Chinese adolescents more vulnerable to depression (Wang et al., 2014; Hollenstein et al., 2015; Fan, 2016; Kloep et al., 2016). It seems that the therapists in our study tailored their interventions in response to this cultural characteristic. They helped the families establish boundary awareness, suggested that overprotective parents give their children more freedom, and encouraged adolescents to focus more on their own interests and social relationships. All of these therapeutic strategies to facilitate the adolescents’ individual differentiation were appreciated by the participants.

With respect to *facilitating emotional expression and flow*, our analysis demonstrated that supportive emotional expression techniques such as listening and empathy could facilitate the expression and relief of the families’ emotions and pain. This is similar to the results of prior studies (Sundet, 2011; Lovgren et al., 2019; Tsvieli et al., 2019). Wang et al.’s studies reported poorer family interpersonal communication in Chinese families with a depressed family member than in Chinese families without depressed family members, and after systematic family therapy, not only were depressive symptoms relieved, but family communication was also improved (Wang et al., 2013; Li et al., 2016). This finding partially supports the interventions of promoting family emotional exchange used by the therapists in our study.

Regarding the fourth theme, *effective systemic therapy techniques* used by the therapists were well-recognized by the families. This finding confirms findings of previous research on the perspective of depressed adolescents. For example, Liu et al. found that metaphors, stories of famous people and similar cases, and spatial metaphors were frequently used by Chinese family therapists to facilitate families’ understanding during successful family therapy (Li et al., 2016). Additionally, previous research has shown that linear and circular questioning was employed by family therapists as a core technique to explore family relations and perspectives (Liu et al., 2013; de Leon, 2018). Moreover, homework has been found to be a frequently used tool to trigger and promote family resources and change (Li et al., 2016).

Our fifth theme, *strategies for dealing with current issues*, indicated the value of the exploration and discussion of the daily challenges confronted by adolescents, especially academic difficulties and crises. The emergence of the subtheme *coping with academic difficulties* may be strongly influenced by the current situation in Chinese society. As mentioned above, China’s senior high school students are facing greater pressure with respect to college entrance examinations, which could trigger depressive or anxious emotions (Hou and Chen, 2016). Indeed, nearly all the adolescents in our study were high school students. Some of them even exhibited school refusal due to academic stress. Most of them benefited from discussions of their academic difficulties with the therapists, which allowed them to clarify their stress and related emotions and explore potential coping strategies that their families could use to handle these difficulties. This finding implies that, in therapy for depressed Chinese adolescents in high school, academic stress is a significant topic. Furthermore, some adolescents in our study reported self-harm and suicidal ideations. Our analysis implied that when the adolescents’ self-harm or suicidal issues emerged, the parents’ expectation for their children’s academic performance would decline. Their concern for children’s mental health increased, while they showed better compliance to psychotherapy. Correspondingly, for these clients with suicidal tendencies, therapists’ respectful, attentive, non-accusatory attitudes and their alliance building and firm prevention efforts were appreciated by the subjects’ families. This humanized, professional style may strengthen the therapeutic alliance and boost the families’ confidence to cope with the crises. It is recommended in future psychotherapy for adolescent depression.

This study has several limitations. First, we did not conduct independent interviews for the parents and children, which made it impossible to distinguish between the perspectives of the children and those of their parents. As demonstrated by prior studies, the perceptions of parents might be different from those of their children (Solomon et al., 2018). Thus, future research analyzing the opinions of parents and children separately and comparing perspectives between the two groups is strongly suggested. Second, our study is an exploratory analysis with a small sample of adolescents in high school. We should be circumspect when extrapolating the results to larger and more diverse populations. Quantitative studies with more clients could be carried out to test the validity of our analysis. Third, all the therapists who delivered interventions in our study were psychiatrists with a background in family therapy. It could be considered a pretreatment characteristic of family therapy and may have led to bias in the results of our analysis. For instance, therapists’ professional background as a psychiatrist may have enhanced the clients’ trust in their therapists. It might indirectly influence the clients’ subjective evaluation of the effects of some interventions received during therapy. Meanwhile, compared with clinical psychologists, psychiatrists may focus more on some medical issues such as the details of clients’ depressive symptoms and the adjustment of their medication. This potential difference in the focus of therapy may also influence the participants’ experience of family therapy. Future research including more therapists with other professional backgrounds is suggested to clarify this potential bias. Fourth, some participants in our study accepted medication and psychotherapy at the same time. Antidepressants may influence adolescents’ experiences of depressive symptoms and family therapy. It may be meaningful to compare the experience of the subjects in the medication group with that of those in the psychotherapy only group in future studies.

### Clinical Implications

The themes that emerged in our study can help clinicians develop targeted family therapeutic strategies for depressed Chinese adolescents. First, our results imply that to help depressed adolescents, therapists should cultivate their professional experience of psychotherapy and psychiatry and their own personality traits, such as patience, affinity, and warmth. Second, in the process of therapy, more attention could be paid to identifying, exploring, and expanding the resources of adolescents and their families using non-authoritative and non-judgment approaches. Third, the analysis highlights the importance of promoting adolescents’ self-differentiation by establishing boundary awareness in the family and enriching safe child-parent attachment by encouraging affective expression among family members and focusing on adolescents’ unmet emotional needs (Diamond et al., 2002; Ewing et al., 2015). As Minuchin et al. (2013) suggested, therapists may need to help families transform their parent-child dualistic interactions into a “close and independent” pattern. Fourth, it seems that circular questioning and behavioral homework still play central roles in exploring and transforming emotions and interpersonal patterns in the families of adolescents. In other words, “questioning is better than the best advice.” Finally, our study implied that to help depressed Chinese teenagers, academic stress is a key topic that cannot be ignored during psychotherapy.

## Conclusion

This study highlights the importance of taking clients’ subjective experiences into consideration when designing therapeutic strategies and methods for families with depressed adolescents. A trustworthy therapeutic alliance with a competent and supportive therapist helped depressed adolescents improve by facilitating the expression of their emotions; exploring family interactions, beliefs, and emotion flows related to their symptoms; promoting their self-development; and activating their resources. The families valued the respectfulness of the therapists and a collaborative and non-authoritative approach. Special attention should also be paid to adolescents’ academic difficulties and crises.

## Data Availability Statement

The datasets generated for this study are available on request to the corresponding author.

## Ethics Statement

The studies involving human participants were reviewed and approved by Ethics committee of the Tongji University and the Shanghai Pudong New Area Mental Health Center. Written informed consent to participate in this study was provided by the participants’ legal guardian/next of kin.

## Author Contributions

JWu, JWa, YW, and YT conducted interviews with the participants and transcribed the data. CG provided assistance with the transcription of the interviews. LL and YbW developed the interview outline, analyzed the data, and took the main responsibility for drafting the manuscript. All authors contributed to the article and approved the submitted version.

## Conflict of Interest

The authors declare that the research was conducted in the absence of any commercial or financial relationships that could be construed as a potential conflict of interest.
